# Effect of Storage Time, Thermocycling And Resin Coating on Durability of Dentin Bonding Systems

**Published:** 2009

**Authors:** Seyed-Mostafa Mousavinasab, Azadeh Farhadi, Mitra Shabanian

**Affiliations:** * Associate Professor, Department of Restorative Dentistry and Torabinejad Dental Research Centre, School of Dentistry, Isfahan University of Medical Sciences, Isfahan, Iran; ** Dentist, Private Practice, Isfahan University of Medical Sciences, Isfahan, Iran; *** Assistant Professor, Department of Restorative Dentistry and Torabinejad Dental Research Centre, School of Dentistry, Isfahan University of Medical Sciences, Isfahan, Iran

**Keywords:** Dental adhesives, shear strength, storage

## Abstract

**Background:**

Along with development of different dental adhesives, concerns about hydrolytic deg-radation of the adhesive components have arisen. The purpose of this study was to evaluate the in vitro influence of thermocycling, water storage and resin coating on the microshear bond strength of total etch and self etch adhesive systems to dentin.

**Methods:**

The superficial coronal dentin of eighty intact third molars were exposed and divided into 5 equal groups. Dental adhesives including Scotch Bond Multi Purpose (SBMP), Single Bond (SB), Clearfil SE Bond (CSE), Prompt L-Pop (PLP), and Prompt L-Pop plus Margin bond (PLPM) were applied according to the manufacturers’ instructions on prepared surfaces in the study groups, respectively. Then composite cylinders were bonded and specimens were divided into two subgroups. One subgroup was stored in water for 24 hours. The second subgroup was subjected to 3000 thermocycle shocks and then was stored in 37°C water for 3 months. Finally, all teeth were subjected to the microshear bond strength test. Data were analyzed using two-way ANOVA and Tukey HSD tests. One specimen similar to each subgroup was also prepared for SEM evaluation.

**Results:**

After one-day storage, the SBMP showed the highest bond strength followed by CSE, PLPM, SB and PLP. After three months storage, the highest bond strength was observed in SBMP followed by PLPM, CSE, SB, and PLP.

**Conclusion:**

SBMP showed the best bond strength while CSE represented acceptable bond durability. Resin coating on PLP improved bond strength and durability.

## Introduction

The long-term durability of bonds between adhesive resin systems and dentin is important for the longevity of bonded restorations. Self-etching adhesives are widely employed, mainly because of their ease of use and low technique sensitivity. However, the longevity of adhesive bonds is still an area of interest in adhesive dentistry. Bond durability of various dental adhesive systems has been the subject of several studies.^1^ In one study, Clearfil Liner Bond II showed stable microtensile bond strength (μTBS) of approximately 19 MPa during the one-year testing period, although SEM examination revealed increasing porosity at the top of the hybrid layer and within the adhesive resin over time.^2^

Koshiro et al showed that even though the bond strengths of both adhesive systems declined over time, the bonding interface using self-etching primers was relatively stable compared to the wet bonding system.^3^ Munck et al in a study concluded that resin bonded to enamel protects the resindentin bond against degradation, while direct exposure to water for 4 years affected bonds produced by two-step total-etch adhesives, a finding which is in agreement with the results of other studies.^4^,^5^

Durability of three simplified systems investigated by Dijken et al revealed that in a period of 24 months, Prompt L-Pop showed a significantly higher cumulative loss rate compared to Clearfil Liner Bond 2 and One Coat Bond.^6^ Investigation of the changes in intact dentin collagen fibrils after water storage using a total etch luting resin (Super-Bond C& B) and a self-etching luting resin (Panavia F 2.0) showed that the top of hybrid layer contained disorganized collagen fibrils from the smear layer, which degraded over time.^7^Other studies have also shown micro-morphological changes in dentin resin bond after varying storage times.^8^–^11^ Armstrong et al also found differences in the bond strength of one-step and two-step adhesive systems with up to 6 months of storage but no differences were noted at 15 months. This may represent common degradative mechanisms.^12^

Some studies have confirmed that simplified bonding procedures do not necessarily provide improved bonding performance, especially in the long term but rather suggest that the resistance of resin dentin bonds to degradation depends on the material.^13^–^15^ It has been speculated that hydrolytic degradation within the hybrid layer gradually increases due to water penetration through nanoleakage channels, resulting in lower bond strengths and interfacial failure after as little as nine months.^16^ It has also been suggested that the bond strength of different solvent-based adhesive systems gradually decreases over time regardless of the variable moisture pattern used for the bonding procedure.

In addition to the effect of the adhesive system, thermocycling can decrease bond durability.^17^,^18^ HEMA-free one-step adhesives are prone to phaseseparation, which may also account for their lower bonding effectiveness.^19^ One study confirms that bonded enamel margins may not maintain the integrity of the resindentin interface created by HEMA-free and HEMA-containing one-step adhesives.^20^

Studies of the effects of copolymer hydrophilicity and temperature on water sorption and solubility characteristics on bond durability indicate that water molecules diffuse through the polymer matrices by binding successively to the polar sites via hydrogen bonding. Such water sorption may determine the durability of resindentin bonds.^21^ Bonding agents containing hydrophilic monomers have been shown to have a negative influence on resin ceramic bond durability.^22^

To understand the relationship between the water absorption and the durability of adhesive strength in the oral cavity, Tanaka et al designed a series of O-methacryloyl-N-acyl tyrosines (MAATY)-2-hydroxyethyl methacrylate (HEMA) bond system samples. They found that preparation of MAATY, which absorbs less water, might improve durability even when immersed in water.^23^ Frankenberger et al showed that Prompt L-Pop, when applied in multiple coats, resulted in bond strengths that were not statistically different from those of Prime & Bond NT, a total-etch adhesive.^24^

Resin coating technique increases durability and bond strength of simplified step adhesives to composite.^25^,^26^ In a study by Carvalho et al, placement of an intermediate layer of a low-viscosity bonding resin between the bonded dentin surface and the resin cements resulted in improved coupling of Panavia F to dentin.^27^ The effectiveness of resin coating has been documented in other studies.^28^–^30^

The aim of this study was to evaluate the effect of storage time and thermocycling on dentin adhesives bond durability and also resin coating technique on it, in an all-in-one dentin bonding system.

## Materials and Methods

In this experimental study eighty caries-free third molars from patients aged 20-30 years old were collected and stored in 0.2% Thymol solution. The teeth were mounted vertically in cold curing acrylic resin (Acropars 200, Iran) using plastic circular molds. Superficial coronal dentin was exposed by horizontally trimming (Krupp Dental Dentarapid GMBH, Fride Krupp GMBH, Krup WIDIA, N; 759 DR2, Germany) the occlusal sur-face of each tooth crown under running water. After trimming, the resulting surfaces were finished using diamond burs (Komet dental Co, 859EF, UK) under running water. The prepared teeth were assigned to five groups of sixteen each.

Scotch Bond Multi Purpose (SBMP), Single Bond (SB), Clearfil SE Bond (CSE) and Prompt L-Pop (PLP) were applied on the prepared surfaces respectively, according to manufacturer instruction (groups 1 to 4).

Finally in group 5 (PLPM), after application of Prompt L-Pop, one layer of Margin bond was applied. The characteristics of used materials are shown in Table 1.

**Table 1:** Characteristics of the tested materials.

Materials	Batch and s Company	Composition	Procedure
Scotch Bond Multi Purpose	70-2010-1608-9	Primer: HEMA/ poly alkenoic copolymer/ water Resin: Bis – GMA/ HEMA	1-Etching: Apply phosphoric acid, wait 15 sec, rinse for 15 sec, dry for 2 sec, leave moist.
	3M ESPE, USA		2-Priming: apply primer and dry gently for 5 sec.
			3-Apply adhesive to tooth.
			4-Light cure for 10 sec.
Adper Single Bond	70-2010-3498-3	Bonding: Bis – GMA/ HEMA poly alkenoic copolymer/poly itaconic dimethacrylate /water / ethanol	1- Etching: apply scotch bond etchant, wait 15 sec, Rinse for 10 sec, blot excess water, Leaving tooth moist.
	3M ESPE, USA		2-Adhesive: Apply 2 consecutive coats, dry gently for 2-5 sec
			3-light cure for 10 sec.
Clearfil SE Bond	1976-WD	Primer: HEMA/ MDP /Hydrophilic DMAV, N-diethanol P-toluidine Adhesive : MDP/Bis GMA/HEMA/Hydrophobic DMA/sillanated colloid silica, N N-Diethanol p- toluidine/camphorquinone	1- Gently air dry surface.
	Kuraray, Japan		2-Apply SE primer for 20 sec, gently air dry to evaporate solvent.
			3-Apply SE- bond & light activate for 10 sec.
Adper Prompt – L Pop	2007-06D	Methacrylated phosphoric acid ester fluoride complex / parabense /water / stabilizer/ photoinitiator	1-Active blister pack by emptying the liquid out of the red blister into yellow blister.
	3M ESPE, USA		2-The activated mixture is applied to tooth substrate with agitation for 15 sec, no rinsing.
			3-Briefly air dry, light activate for 10 sec using VLC unit.
Margin bond	MB 004	Composition : BIs GMA/ Bis EMA / TEGDMA	1, 2, 3 - steps like as prompt L- pop group.
	Coltene, Switzerland		4- Apply a thin layer of margin bond and light activate for 20 sec.
Filtek Z 250	70-2010-2565-0 3M - USA	Monomer : Bis- GMA/ TEGDMA Filler : Barium glass	1- The plastic mold filled using resin composite
			2-The filled mold inserted on prepared surface and cured for 40 sec.

One mm internal diameter plastic cylindrical molds were filled with resin composite (Filtek Z250, 3M ESPE, USA) and attached to conditioned dentin surfaces and cured for forty seconds using a tested LED light source (Bluphase, Ivoclar Vivadent, Germany). The teeth in each group were divided into two subgroups. One subgroup was subjected to the micro shear test after 24 hours storage in distilled water at 37°, the second subgroup was tested after subjecting to 3000 thermocycle shocks (Vafaii Iindusrial Corporation, Iran) and storage in distilled water for three months. The teeth were subjected to μSBS using a universal testing machine (Dartec, England) at 1mm/min speed. The patterns of fracture were shown using a stereomicroscope (MBC-1O, number: N9116734, Russia) at 28X magnification and were divided into the following categories: adhesive, cohesive in dentin, cohesive in restorative material and mixed failure.

In addition to subjecting samples to micro-shear bond strength tests, one additional sample was prepared in each group for SEM evaluation. The prepared teeth were sectioned perpendicular to the composite interface and then prepared for SEM investigation (Philips XL30, Philips, Eindhoven, Netherlands).

Data were analyzed using SPSS software. Two-way ANOVA was used to compare differences in mean micro shear bond strength values of the groups. Independent sample t-test was used to compare values of each bonding system group stored for one day or three months and finally, the Tukey’s test was used to compare the differences between paired groups.

## Results

Data for the bond strength tests are shown in Table 2. Based on independent sample t-test, the differences between groups with different storage times were only significant for SB and PLP (p < 0.05).

**Table 2:** Mean (SD) bond strength of the tested groups (MPa).

Groups Storage time	SBMP	SB	CSE	PLP	PLPM
1d	40.78 (4.08)	25.62(5.75)	33.25(5.80)	11.75(4.68)	34.00(5.58)
3m	42.25(6.08)	14.75(6.34)	31.5(7.65)	11.12(6.75)	33.25(6.11)

1d: 24 hours storage time, 3m: 3 months storage time, SBMP: Scotch Bond Multi Purpose, SB: Single Bond, CSE: Clearfield SE bond PLP: Prompt L-Pop and PLPM: Prompt L-Pop plus Margin Bond.

SBMP bond strength (40.87 MP) was significantly higher than the other tested groups after one-day storage time. The difference between CSEB (28.4 MPa) and PLPM (29.3 MPa) was not significant, but the differences between CSE, PLPM and the other groups were significant. The difference between SB (25.62 MPa) and PLP (the lowest value) with the other groups was also significant.

After 3 months storage time, the highest bond strength value related to SBMP (42.25MPa) and its difference with other groups was significant. The difference between SB (14.7MPa) and PLP (11.1 MPa) was not significant. The moderate strengths were related to SE (31.5 MPa) and PLPM (33.2 MPa) and there was no significant difference between them. But there were significant differences between CSE or PLPM with the other groups.

**Figure 1:**
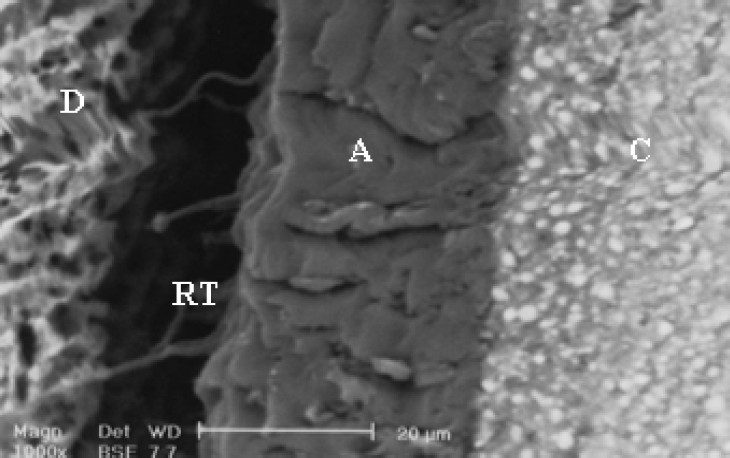
SEM observation of SBMP after one-day storage time. A (adhesive), C (resin composite) D (dentin), RT (resin tags)

**Figure 2:**
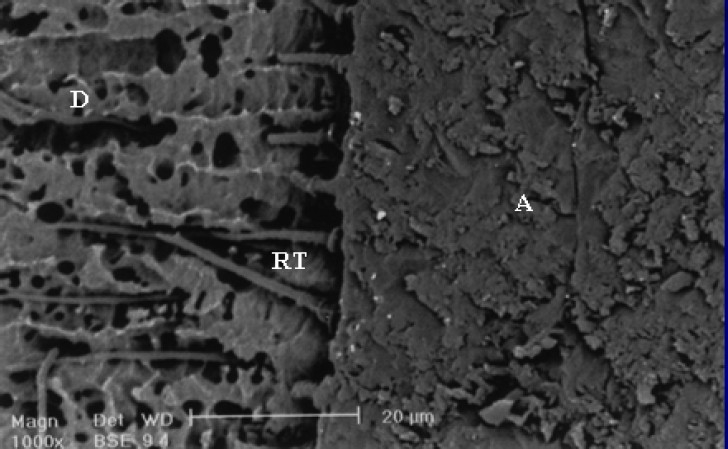
SEM observation of SBMP after three-month storage time. A(adhesive), C(resin composite) D (dentin), RT(resin tags)

SEM evaluation showed formation of different hybrid layers and the occurrence of changes for some tested adhesive systems stored for one day or three months (Figures 1–2). After three months storage, the bond strength of resin dentin using SBMP remained stable. The fracture mode in samples stored for 24 hours and three months storage time are summarized in table 3.

**Table 3:** Fracture mode distribution of the tested groups.

Groups	Mixed Fracture (%)	Adhesive Fracture (%)	Cohesive Fracture(%)in resin composite	Cohesive Fracture(%) in dentin	Premature Fracture (%)
SBMP.1d	37.5	12.5	0	50	0
SB.1d	50	25	12.5	12.5	0
CSE. 1d	37.5	25	0	37.5	0
PLP. 1d	12.5	75	12.5	0	0
PLPM. 1d	37.5	62.5	0	0	0
SBMP.3m	12.5	50	0	37.5	0
SB.3m	12.5	75	0	0	0
CSE.3m	0	50	12.5	37.5	0
PLP.3m	0	100	0	0	0
PLPM.3m	0	75	0	25	0

1d: 24 hours storage time, 3m: 3 months storage time, SBMP: Scotch Bond Multi Purpose, SB: Single Bond, CSE: Clearfield SE bon, PLP: Prompt L-Pop and PLPM: Prompt L-Pop plus Margin Bond.

## Discussion

The sealing ability of dentin bonding agents is a determinant factor in the durability of adhesive restorations, especially in dentinal margins. Dentin is naturally wet and it interferes with the bonding process to dentin and with its durability. To overcome these problems, hydrophobichydrophilic dentin bonding agents have been designed, but hydrophilicity will lead to the attraction and accumulation of water in the hybrid layer and finally to its degradation.^31^

In the present study, SBMP showed higher bond strength compared to other bonding agents both after one day and three months storage. This finding suggests that clinicians may wish to prefer the multi-step dentin bonding agents to the one step type^32^ because of its lesser technique sensitivity^4^ Another important factor maybe formation of the desired hybrid layer following the specially designed primer containing HEMA in water and the presence of a glass-filled viscous solvent free resin^11^

The single bond agent contains HEMA, water, and ethanol as solvent in its composition. HEMA lowers vapor pressure, which affects Bis-GMA monomer penetration into the demineralized area. The remaining water interferes with the polymerization of the bonding agent and can be the cause of a decrease in bond strength as shown in this study.^33^ Formation of water trees and the improper use of wet bonding technique can be other factors involved in lower bond strength with single bond.^13^,^34^

After mixing adhesives and self-etching primer in the 2-step single-bottle, the adhesives are more permeable and hence absorb more water over time than previous generations of adhesives. The most recent single-step self-etching adhesives are even more hydrophilic and, as a result, more permeable to water derived from the underlying bonded dentin.^35^

A 44% reduction in bond strength after three months storage for the teeth bonded with single bond can be attributed to the higher concentration of primer/adhesive, following simplification in the adhesive system and production of a more sensi-tive hybrid layer during aging. Remaining solvent or water on the surface can lead to the degradation and leaching out of the resin during storage.^4^ Hydrophilic dentin adhesives polymerized in thin films are prone to water loss due to evaporation. This probably accounts for the water droplets seen on the surface of vital-bonded dentin after the ap-plication of simplified dentin adhesives.^2^ SEM results showed a more uniform and complete penetration of the Scotch Bond into the collagen fibers compared to the Single Bond adhesive, which may explain its easer degradation.

CSE as a mild two-step self-etching process, showed a higher bond strength compared to SB and PLP and a lower related bond strength compared to SBMP. This finding is in accordance with the results of other studies and can be attributed to the presence of 10 MDP (a functional hydrophil monomer) in the CSE. Higher chemical bond formation to hydroxyapatite compared to other functional molecules and hydrolytic stability are other related factors.^13^,^33^,^36^ Presence of 10 MDP functional monomers and filler particles in CSE and formation of a relatively thick layer that serves as an elastic buffer zone during polymerization of resin composite ensure bond durability.^37^

Good wetability and penetration in dentin bonding agents containing filler particles (like CSE) with the presence of elastomeric dimethacrylate can function to compensate for the polymerization shrinkage of composite resin.^33^ Compared to dentin bonding agents including separate acidic conditioner, the made CSE hybrid layer is shallower. Acidic monomers assure coordinate etching and penetration of primer that prevents collapse of the collagen fibers.^1^ Bond strength during three-month storage time in CSE samples was constant and the changes observed in SEM findings after one day and three-month storage were similar.

Yoshida states that mild self-etch adhesives demineralize dentin only partially, leaving hydroxyapatite around collagen within a submicron hybrid layer. He hypothesizes that this residual hydroxyapatite may serve as a receptor for chemical interaction with the functional monomer and, subsequently, contributes to adhesive performance in addition to micro-mechanical hybridization.^38^

In this study, the low bond strength gained with PLP is an indication of the low bond quality produced by this bonding system, which showed a significant decrease in strength after the three-month storage period. This finding is in agreement with findings reported earlier^24^ and suggests that incorporation of hydrophilic monomers with the hydrophobic monomer in one solution adversely affects bonding agent function.^33^

For PLP, a 37% decrease in bond strength after the three-month storage period can be related to low viscosity, more hydrophilic composition, and the inhibitory effect of oxygen. The solvent in PLP is merely water compared to the presence of water and ethanol in CSEB as solvent. The higher amount of water in PLP affects its bonding durability.^39^ In addition, one-step self-etching systems act like a permeable membrane after polymerization. Higher acidity and the hydrophilic nature of acidic monomers increase the risk of hydrolytic degradation.^40^

SEM evaluation showed that in contrast to the three-step adhesives, the PLP hybrid layer was very thin as indicated by Frankenberger who described an inconsistent hybrid layer for PLP specimens.^24^ The combination of PLP plus a hydrophobic layer (margin bond) showed results similar to a CSE with good bond durability after a three-month storage time. Using a hydrophobic layer decreases the degradation of the bond, overcoming the drawback of PLP bond durability as studies show that using more hydrophobic monomers in adhesive formulation can prolong bond durability.^17^

Water sorption and the solubility of an adhesive are determinant factors in bond quality and durability and the final success of a restorative material.^41^ The positive relationship between maximum water uptake and copolymer hydrophilicity suggests that water molecules diffuse through the polymer matrices by binding successively to the polar sites via hydrogen bonding. Such water sorption may determine the durability of resindentin bonds,^42^ as indicated by the differing percent of higher cohesive dentinal failure in samples stored for one day compared to samples stored for three months in all the bonding agent systems tested in the present study.

In resin composite restorations, bonded using acidic adhesives, an extremely acidic monomer comes in contact with adjacent composite resin, which affects bond durability. In these cases, “water trees” that represent channels of increased permeability with the polymerized adhesive layer are also observed in the one-step adhesives. In the cases involving CSE and SBMP, however, an intermediary hydrophobic and less acidic resin layer is located between primer and composite layer, which controls destructive chemical reactions.^43^–^44^ Tay et al found that application of chemical co-initiator (Bondlink) as a medium layer on cured single step adhesive further improved the bond strength compared to a control group.^45^ This is the basis of the resin coating technique described in some studies as a way to enhance the bond strength of resin cement to dentin.^28^–^30^Application of an intermediate resin layer produces a relatively thick layer between dentin and the polymerizing composite.

This can compensate for polymerization stresses, transfer and dissipate thermal, hygroscopic, and occlusal stresses, and counteract the oxygen inhi-bition effect.

**Figure 3:**
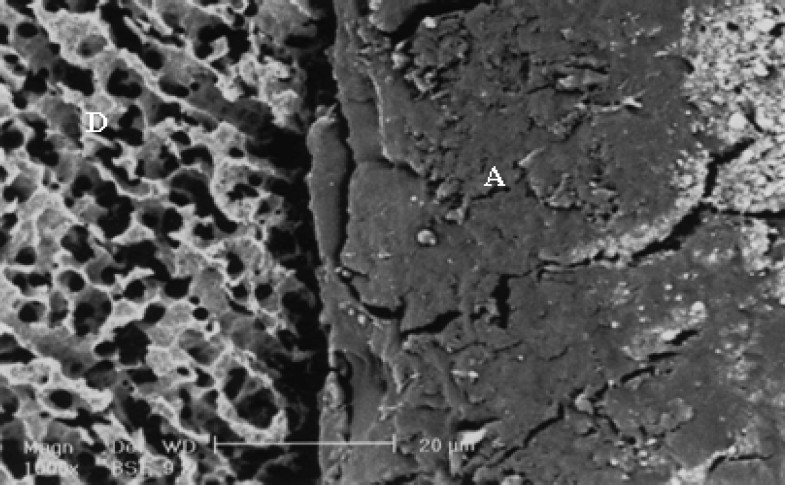
SEM observation of PLPM after one-day storage time. A (adhesive), C (resin composite), D (dentin).

**Figure 4:**
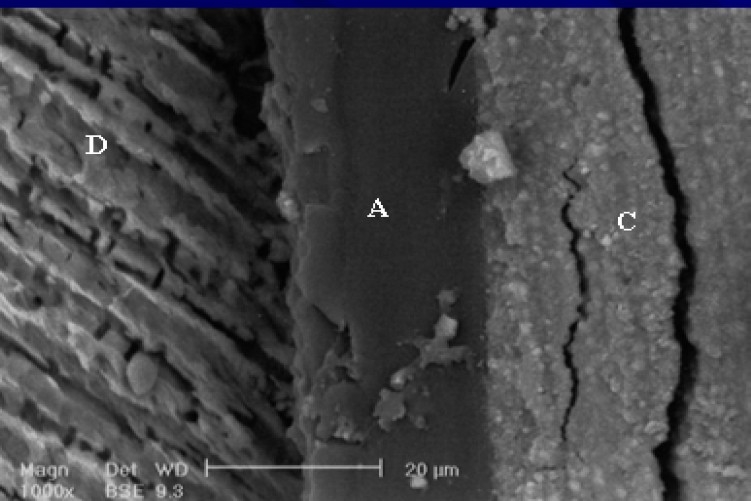
SEM observation of PLPM after three-month storage time. A (adhesive), C (resin composite), D (dentin). There is no sign of disintegration after storage time.

## Conclusion

Tested three-step total etch adhesive in the present study showed the best bond strength and durability.

Two-step self etch adhesives used in this study represented acceptable bond durability. Application of a hydrophobic resin layer (resin coating) on the simplified dentin adhesive used in this study had a positive effect on bond strength and durability.

## References

[1] Amaral FL, Colucci V, Palma-Dibb RG, Corona SA (2007). Assessment of in vitro methods used to promote adhesive interface degradation: a critical review. J Esthet Restor Dent.

[2] Sano H, Yoshikawa T, Pereira PN, Kanemura N, Morigami M, Tagami J (1999). Long-term durability of dentin bonds made with a self-etching primer, in vivo. J Dent Res.

[3] Koshiro K, Inoue S, Tanaka T, Koase K, Fujita M, Hashimoto M (2004). In vivo degradation of resindentin bonds produced by a self-etch vs. a total-etch adhesive system. Eur J Oral Sci.

[4] De Munck J, Van Meerbeek B, Yoshida Y, Inoue S, Vargas M, Suzuki K (2003). Four-year water degradation of total-etch adhesives bonded to dentin. J Dent Res.

[5] Gamborgi GP, Loguercio AD, Reis A (2007). Influence of enamel border and regional variability on durability of resindentin bonds. J Dent.

[6] van Dijken JW (2004). Durability of three simplified adhesive systems in Class V non-carious cervical dentin lesions. Am J Dent.

[7] Yang B, Adelung R, Ludwig K, Bossmann K, Pashley DH, Kern M (2005). Effect of structural change of collagen fibrils on the durability of dentin bonding. Biomaterials.

[8] Hashimoto M, Ohno H, Kaga M, Endo K, Sano H, Oguchi H (2001). Resin-tooth adhesive interfaces after long-term function. Am J Dent.

[9] Hashimoto M, Ohno H, Sano H, Tay FR, Kaga M, Kudou Y (2002). Micromorphological changes in resindentin bonds after 1 year of water storage. J Biomed Mater Res.

[10] Takahashi A, Inoue S, Kawamoto C, Ominato R, Tanaka T, Sato Y (2002). In vivo long-term durability of the bond to dentin using two adhesive systems. J Adhes Dent.

[11] Hashimoto M, Fujita S, Kaga M, Yawaka Y (2007). In vitro durability of one-bottle resin adhesives bonded to dentin. Dent Mater J.

[12] Armstrong SR, Vargas MA, Fang Q, Laffoon JE (2003). Microtensile bond strength of a total-etch 3-step, total-etch 2-step, self-etch 2-step, and a self-etch 1-step dentin bonding system through 15-month water storage. J Adhes Dent.

[13] Shirai K, De Munck J, Yoshida Y, Inoue S, Lambrechts P, Suzuki K (2005). Effect of cavity configuration and aging on the bonding effectiveness of six adhesives to dentin. Dent Mater.

[14] Osorio R, Pisani-Proenca J, Erhardt MC, Osorio E, Aguilera FS, Tay FR (2008). Resistance of ten contemporary adhesives to resindentine bond degradation. J Dent.

[15] Nam KY, Kim JB, Jang BC, Kwon TY, Kim KH (2007). Effects of dentin bonding agents on bonding durability of a flowable composite to dentin. Dent Mater J.

[16] Okuda M, Pereira PN, Nakajima M, Tagami J, Pashley DH (2002). Long-term durability of resin dentin interface: nanoleakage vs. microtensile bond strength. Oper Dent.

[17] Reis A, Loguercio AD, Carvalho RM, Grande RH (2004). Durability of resin dentin interfaces: effects of surface moisture and adhesive solvent component. Dent Mater.

[18] Huang MS, Li MT, Huang FM, Ding SJ (2004). The effect of thermocycling and dentine pre-treatment on the durability of the bond between composite resin and dentine. J Oral Rehabil.

[19] Van Meerbeek B, Van Landuyt K, De Munck J, Hashimoto M, Peumans M, Lambrechts P (2005). Technique-sensitivity of contemporary adhesives. Dent Mater J.

[20] Torkabadi S, Nakajima M, Ikeda M, Foxton RM, Tagami J (2008). Bonding durability of HEMA-free and HEMA-containing one-step adhesives to dentine surrounded by bonded enamel. J Dent.

[21] Yiu CK, King NM, Carrilho MR, Sauro S, Rueggeberg FA, Prati C (2006). Effect of resin hydrophilicity and temperature on water sorption of dental adhesive resins. Biomaterials.

[22] El Zohairy AA, De Gee AJ, Hassan FM, Feilzer AJ (2004). The effect of adhesives with various degrees of hydrophilicity on resin ceramic bond durability. Dent Mater.

[23] Tanaka J, Ishikawa K, Yatani H, Yamashita A, Suzuki K (1999). Correlation of dentin bond durability with water absorption of bonding layer. Dent Mater J.

[24] Frankenberger R, Perdigao J, Rosa BT, Lopes M (2001). “No-bottle” vs “multi-bottle” dentin adhesives--a microtensile bond strength and morphological study. Dent Mater.

[25] Jayasooriya PR, Pereira PN, Nikaido T, Burrow MF, Tagami J (2003). The effect of a “resin coating” on the interfacial adaptation of composite inlays. Oper Dent.

[26] Nikaido T, Nakaoki Y, Ogata M, Foxton R, Tagami J (2003). The resincoating technique. Effect of a singlestep bonding system on dentin bond strengths. J Adhes Dent.

[27] Carvalho RM, Pegoraro TA, Tay FR, Pegoraro LF, Silva NR, Pashley DH (2004). Adhesive permeability affects coupling of resin cements that utilise selfetching primers to dentine. J Dent.

[28] Jayasooriya PR, Pereira PN, Nikaido T, Tagami J (2003). Efficacy of a resin coating on bond strengths of resin cement to dentin. J Esthet Restor Dent.

[29] Kosaka S, Kajihara H, Kurashige H, Tanaka T (2005). Effect of resin coating as a means of preventing marginal leakage beneath full cast crowns. Dent Mater J.

[30] Kitasako Y, Burrow MF, Nikaido T, Tagami J (2002). Effect of resin-coating technique on dentin tensile bond strengths over 3 years. J Esthet Restor Dent.

[31] Sano H, Shono T, Takatsu T, Hosoda H (1994). Microporus dentin zone beneath resin-impregnated layer. Oper Dent.

[32] Summitt JB, Robbins JW, Hilton TJ, Schwartz RS, Santos JR (2006). Fundamentals of Operative Dentistry: A Contemporary Approach.

[33] Li H, Burrow MF, Tyas MJ (2000). Nanoleakage patterns of four dentin bonding systems. Dent Mater.

[34] Tay FR, Pashley DH (2003). Water treeing--a potential mechanism for degradation of dentin adhesives. Am J Dent.

[35] Tay FR, Pashley DH (2003). Have dentin adhesives become too hydrophilic?. J Can Dent Assoc.

[36] Kubo S, Yokota H, Sata Y, Hayashi Y (2001). Microleakage of self-etching primers after thermal and flexural load cycling. Am J Dent.

[37] Inoue S, Koshiro K, Yoshida Y, De Munck J, Nagakane K, Suzuki K (2005). Hydrolytic stability of self-etch adhesives bonded to dentin. J Dent Res.

[38] Yoshida Y, Nagakane K, Fukuda R, Nakayama Y, Okazaki M, Shintani H (2004). Comparative study on adhesive performance of functional monomers. J Dent Res.

[39] Tay FR, Pashley DH, Garcia-Godoy F, Yiu CK (2004). Single-step, self-etch adhesives behave as permeable membranes after polymerization. Part II. Silver tracer penetration evidence. Am J Dent.

[40] van Dijken JW (2004). Durability of three simplified adhe-sive systems in Class V non-carious cervical dentin lesions. Am J Dent.

[41] Mortier E, Gerdolle DA, Jacquot B, Panighi MM (2004). Importance of water sorption and solubility studies for couple bonding agent--resin-based filling material. Oper Dent.

[42] Yiu CK, King NM, Carrilho MR, Sauro S, Rueggeberg FA, Prati C (2006). Effect of resin hydrophilicity and temperature on water sorption of dental adhesive resins. Biomaterials.

[43] Tay FR, King NM, Suh BI, Pashley DH (2001). Effect of delayed activation of light-cured resin composites on bonding of all-in-one adhesives. J Adhes Dent.

[44] Cheong C, King NM, Pashley DH, Ferrari M, Toledano M, Tay FR (2003). Incompatibility of self-etch adhesives with chemical/dual-cured composites: two-step vs one-step systems. Oper Dent.

[45] Tay FR, Pashley DH, Yiu CK, Sanares AM, Wei SH (2003). Factors contributing to the incompatibility between simplified-step adhesives and chemicallycured or dual-cured composites. Part I. Single-step self-etching adhesive. J Adhes Dent.

